# Xuezhikang Therapy Increases miR-33 Expression in Patients with Low HDL-C Levels

**DOI:** 10.1155/2014/781780

**Published:** 2014-01-23

**Authors:** Ruihua Cao, Yongyi Bai, Lan Sun, Jin Zheng, Mian Zu, Guanhua Du, Ping Ye

**Affiliations:** ^1^Department of Geriatric Cardiology, Chinese PLA General Hospital, No. 28 Fuxing Road, Beijing 100853, China; ^2^National Center for Pharmaceutical Screening, Institute of Materia Medica, Chinese Academy of Medical Sciences and Peking Union Medical College, Beijing 100050, China

## Abstract

*Background*. MicroRNA-33a and -b (miR-33a/b) have been revealed to be posttranscriptional regulators of HDL metabolism. Xuezhikang (XZK) is a marked natural HDL-raising polypill. We aim to evaluate the effects of XZK on the expression of circulating miR-33a/b in patients with low plasma HDL-C levels. *Methods*. A total of 42 participating patients with low baseline levels of HDL cholesterol were assigned to receive an XZK capsule, 600 mg twice daily for 6 months. The expression of circulating miR-33a/b was detected at baseline and after XZK therapy measured with quantitative reverse-transcription (RT) polymerase chain reaction (PCR). *Results*. The mean (SD) HDL-C level after XZK treatment was 1.19 (0.13) mmol/L, representing an increase of 11.2% from baseline (*P* < 0.001). Q-PCR analysis of plasma miRNAs revealed an increase in relative miR-33a/b expression with XZK treatment. The miR-33a expression was raised from 0.81 to 1.73 (*P* = 0.012); miR-33b expression was increased from 1.2 to 2.75 (*P* < 0.001). The changes of miR-33a and miR-33b were inversely related to the posttreatment LDL-C levels (*r* = −0.37, *P* = 0.019; *r* = −0.33, *P* = 0.035, resp.). *Conclusion*. In patients with low HDL-C levels, XZK therapy raised plasma levels of miR-33a and miR-33b, which may inhibit cellular cholesterol export and limit the HDL-raising effect of XZK.

## 1. Introduction

Epidemiologic studies have demonstrated that levels of high-density lipoprotein (HDL) cholesterol, independent of low-density lipoprotein (LDL) cholesterol, are inversely related to cardiovascular risk [1]. Raising levels of HDL cholesterol has been supposed to be an effective therapy for the treatment of atherosclerosis [2, 3]. However, given the complexity of HDL, our understanding of the mechanisms that contribute to HDL metabolism remains incomplete.

MicroRNAs (miRNAs) have emerged as important posttranscriptional regulators of lipid metabolism and therefore a new class of targets for therapeutic intervention [4]. Recently, microRNA-33a and -b (miR-33a/b), intronic miRNAs located within the *SREBF2* and *SREBF1*, respectively, have been revealed to suppress expression of the cholesterol transporter ABC transporter A1 (ABCA1), a key regulator of HDL synthesis [5, 6]. Studies in cell-lines and animal models found that miR-33a/b suppress expression of ABCA1 and lowers HDL levels, while mechanisms that inhibit miR-33 increase ABCA1 and HDL levels [5–7]. Recent advances have demonstrated that some miRNAs can be detected in circulating blood and that these circulating miRNAs might therefore be useful as disease biomarkers [8]. However, whether miR-33a/b can be detected in human plasma has not been reported. The effect of lipid-modulating treatment on the expression of miR-33a/b is also unclear.

Xuezhikang (XZK), an extract of Cholestin, has been approved by the US Food and Drug Administration as a Chinese red-yeast rice dietary supplement. It is composed of a family of natural statins, unsaturated fatty acids, and other substances and has been demonstrated to be a marked natural lipid-modulating polypill [9, 10]. However, the potential mechanisms of HDL-raising effect of XZK have not been completely understood. Whether circulating miR-33a/b levels were changed by XZK treatment requires further investigation.

In the present study, we aim to evaluate the effects of XZK therapy on the expression of circulating miR-33a/b in patients with low plasma HDL-C levels.

## 2. Methods

### 2.1. Selection of Study Subjects

Between September 1, 2010, and June 30, 2011, patients were screened and enrolled from the Pingguoyuan area of the Shijingshan district, a metropolitan area of Beijing, China. After signing informed written consent, eligible potential participants were screened for low plasma HDL-C levels. Initially, 150 participants signed informed consent to be screened. Eighteen subjects with bedridden status, mental illness, and severe systemic diseases were excluded from the analysis.

Clinical data collection and biomarker variable determination were performed in 132 subjects. Among them, a total of 42 subjects with low baseline levels of HDL cholesterol were eligible for analysis. All eligible patients had low baseline levels of HDL cholesterol (<40 mg per deciliter (1.03 mmol per liter) for men; <50 mg per deciliter (1.29 mmol per liter) for women), elevated triglyceride levels (150 to 400 mg per deciliter (1.69 to 4.52 mmol per liter)), and LDL cholesterol levels lower than 180 mg per deciliter (4.65 mmol per liter) [3]. All patients were not receiving lipid-lowering therapy for more than 3 months during the previous 12 months. Patients treated with any lipid-lowering medication within the previous 4 weeks required a 4-week washout period before enrollment to obtain accurate baseline lipid values. The study was approved by the ethics committee of the Chinese People's Liberation Army (PLA) General Hospital, and each participant provided written informed consent.

### 2.2. Selection of Regimens

Because all enrolled patients had established dyslipidemia (low HDL-C levels) and because other trials had demonstrated substantially improved outcomes with XZK therapy, it was deemed ethically unacceptable to randomize patients to placebo or no treatment. Accordingly, all adherent individuals who did not have major clinical events or other serious medical conditions during the run-in were assigned to receive an XZK capsule, 600 mg twice daily (Beijing WBL Peking University Biotech Co., Ltd., Beijing, China) for 6 months.

### 2.3. Laboratory Tests

Blood samples were collected after overnight fasting and serum/plasma aliquots were frozen at −80°C until assays were performed. Concentrations of serum lipid, glucose, liver enzyme, creatinine, and creatine kinase levels were determined using the Roche enzymatic assays (Roche Diagnostics GmbH, Mannheim, Germany) on a Roche autoanalyser (Roche Diagnostics, Indianapolis, Indiana). All tests were performed by well-trained personnel blinded to clinical data.

### 2.4. RNA Preparation and qRT-PCR Analysis

Total plasma RNA was isolated from plasma samples with the TRI Reagent BD (Sigma Aldrich, St. Louis, MO) according to the manufacturer's instructions. Using the All-in-One miRNA qRT-PCR Detection Kit (GeneCopoeia, Rockville, Md, USA), the expression of miR-33a/b was measured with quantitative reverse-transcription (RT) polymerase chain reaction (PCR), according to the manufacturer's instructions. Quantitative PCR was performed by Bio-Rad Real-Time PCR Detection System (Bio-Rad, Hercules, CA).

### 2.5. Statistical Analysis

Continuous variables are presented as mean ± standard deviation (SD) or median (with interquartile range); dichotomous variables are presented as numbers and percentages. Demographic and laboratory characteristics were calculated at baseline and follow-up for all patients completing the trial. Paired *t*-test was used for analysis of the percentage of change in miR-33a/b and lipid values. The Wilcoxon signed rank test was used for continuous variables that were not normally distributed. Pearson's correlation coefficient (*r*) was used to measure the strength of the association between continuous variables. All statistical analyses were performed using Stata software (version 11.0; Stata Corporation, College Station, TX). A 2-sided value of *P* < 0.05 was considered significant.

## 3. Results

### 3.1. Baseline Characteristics

Of the initially enrolled 42 patients, two were lost after 6 months of follow-up. Thus, eventually a total of 40 subjects (mean age 58.7, range 41 to 77 years; 75% were women) were eligible for analysis. Baseline demographic characteristics for the 40 patients completing the trial are summarized in Table 1.

### 3.2. Effects of XZK Treatment on Lipid Profiles

Table 2 summarizes lipid profiles obtained during the study of patients completing the trial. The mean (SD) HDL-C level after XZK treatment was 1.19 (0.13) mmol/L, representing an increase of 11.2% from baseline (*P* < 0.001). The mean (SD) LDL-C level of posttreatment was 2.86 (0.48) mmol/L, a 14.6% reduction from baseline (*P* < 0.001). The median (interquartile range) TG level after treatment of XZK was 2.21 (1.39, 2.80) mmol/L, a 22.5% reduction from baseline (*P* < 0.001). The mean (SD) LDL-C/HDL-C ratio was raised from 0.33 to 0.47 (*P* < 0.001).

### 3.3. Effects of XZK Treatment on Relative miR-33a/b Expression

**Figure 1:**
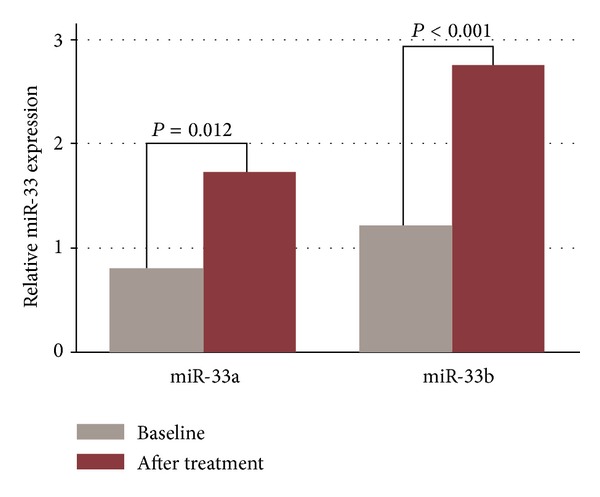
Quantitative real-time fluorescence polymerase chain reaction (QRT-PCR) analysis of miR-33a and miR-33b expression at baseline and after Xuezhikang treatment. Relative expressions of miR-33a/b are raised after Xuezhikang treatment.

Q-PCR analysis of plasma miRNAs revealed an increase in relative miR-33a and -b expression with XZK treatment (Figure 1). The miR-33a expression was raised from 0.81 to 1.73 (*P* = 0.012); miR-33b expression was increased from 1.2 to 2.75 (*P* < 0.001). The changes of miR-33a and miR-33b were inversely related to the aftertreatment LDL-C levels (*r* = −0.37, *P* = 0.019; *r* = −0.33, *P* = 0.035, resp.).

## 4. Discussion

The present study demonstrated for the first time that (1) miR-33a and miR-33b, endogenous miRNAs involved in HDL metabolism, could be detected in human plasma and (2) plasma levels of miR-33a and miR-33b were significantly increased by XZK treatment; changes of miR-33a/b were inversely related to after-treatment LDL-C levels.

miRNAs comprise a class of small, noncoding RNAs that are generally considered to act as intracellular endogenous RNAs to control gene expression on a posttranslational level [11]. Accumulating experimental evidence shows that miRNAs regulate cellular apoptosis, proliferation, differentiation, and migration [12]. Dysregulation of intracellular miRNA expression has been described in various diseases, including HDL metabolism [4].

Rayner et al. firstly reported that miR-33 regulates both HDL biogenesis in the liver and cellular cholesterol efflux [5]. In mouse and human cells, they found that miR-33 inhibits the expression of ABCA1, thereby attenuating cholesterol efflux to apolipoprotein A1 and reducing circulating HDL levels. Conversely, silencing of miR-33 in vivo increases hepatic expression of ABCA1 and plasma HDL levels. Subsequent studies in mice suggest that antagonizing miR-33a may be an effective strategy for raising plasma HDL levels and providing protection against atherosclerosis; however, extrapolating these findings to humans is complicated by the fact that mice lack miR-33b, which is present only in the *SREBF1* gene of medium and large mammals [6, 7].

Despite these findings in cell-lines and animal models, whether miR-33a/b can be detected in human plasma has not been reported. Recently miRNAs circulating in blood have attracted considerable attention [13]. Plasma miRNAs have been reported to be sensitive and specific biomarkers of various tissue injuries and pathological conditions [14–16]. In the present study, for the first time, we found that miR-33a and miR-33b could be detected in human plasma, suggesting that these circulating miRNAs might therefore be useful as biomarkers and might prove useful to monitor status of lipid metabolism.

In this study, in patients with low HDL-C levels, we found that XZK therapy raised HDL-C, which was in line with previously published trials. However, we found that XZK treatment significantly increased plasma levels of miR-33a and miR-33b, which may decrease the expression of ABCA1 and thereby attenuate cholesterol efflux to apolipoprotein A1.

Moreover, we demonstrated that changes of miR-33a/b were inversely related to the reduction of LDL-C levels. It has been reported that [5], in mouse peritoneal macrophages, depletion of cholesterol with simvastatin showed robust upregulation of both miR-33 and *SREBF2*.

Taken together, we propose that XZK treatment raises miR-33a/b expression via a negative feedback loop triggered by reduction of the cholesterol content of the cell (representing reduced LDL-C levels); the upregulation of miR-33a/b inhibits cellular cholesterol export, which may limit the HDL-raising effect of XZK and partly impair the functionality of HDL cholesterol. The possibility that the HDL cholesterol produced by XZK might be dysfunctional deserves careful consideration.

Our findings also suggest that recent failures of drugs that raised HDL-C without reducing cardiovascular disease events or atherosclerosis may partially attribute to the posttranscriptionally effects of miR-33a/b on the functionality of HDL cholesterol.

### 4.1. Limitations

Several limitations of the present study need to be considered. First is the absence of the parallel control group. Because it was deemed ethically unacceptable to administer placebo to patients with established dyslipidemia (low HDL-C levels), we could not include a control group who received either placebo or no treatment. We compensated for the absence of placebo controls by blinding date information on collection of blood sample and resequencing the examinations to eliminate observer bias in interpretation. Secondly, the relatively small sample size is a limitation of the present study. Future research efforts should concentrate on higher-quality and more rigorous trials with larger sample to verify findings of the present study.

## 5. Conclusions

In patients with low HDL-C levels, XZK therapy raised plasma levels of miR-33a and miR-33b, which may inhibit cellular cholesterol export and limit the HDL-raising effect of XZK.

## Figures and Tables

**Table 1:** Baseline patient characteristics.

Characteristics	*N* = 40
Age, y	58.7 ± 10.6
Male gender, number (%)	10 (25)
BMI, kg/m^2^	28.1 ± 4.8
SBP, mm Hg	138 ± 15
DBP, mm Hg	87 ± 9
TC, mmol/L	5.54 ± 0.76
TG, mmol/L^†^	2.85 (2.16, 3.97)
HDL-C, mmol/L	1.07 ± 0.13
LDL-C, mmol/L	3.35 ± 0.72
Fasting glucose, mmol/L	5.4 ± 1.7
Hypertension, number (%)	24 (60)

Age, body mass index (BMI), systolic blood pressure (SBP), diastolic blood pressure (DBP), total plasma cholesterol (TC), high-density lipoprotein cholesterol (HDL-C), low-density lipoprotein cholesterol (LDL-C), and fasting glucose values are given as mean ± standard deviation.

^†^Triglycerides (TG), values as median (quartile 1, quartile 3).

**Table 2:** Changes of lipid profiles after Xuezhikang treatment.

Lipid profiles	Baseline		Aftertreatment		Percent change	*P* value
	Mean (SD)	Median (IQR)	Mean (SD)	Median (IQR)		
TC, mmol/L	5.54 (0.76)	5.57 (4.93–6.07)	4.96 (0.63)	4.90 (4.44–5.96)	−10.5%	<0.001*
HDL-C, mmol/L	1.07 (0.13)	1.11 (0.95–1.18)	1.19 (0.13)	1.19 (1.12–1.30)	11.2%	<0.001*
LDL-C, mmol/L	3.35 (0.72)	3.38 (2.85–3.98)	2.86 (0.48)	2.86 (2.51–3.12)	−14.6%	<0.001*
HDL-C/LDL-C ratio	0.33 (0.07)	0.30 (0.29–0.36)	0.43 (0.07)	0.43 (0.39–0.47)	30.3%	<0.001*
TG, mmol/L	3.32 (1.92)	2.85 (2.16–3.97)	2.21 (0.94)	2.21 (1.39–2.80)	−22.5%	<0.001^†^

SD: standard deviation; IQR: interquartile range; TC: total plasma cholesterol; HDL-C: high-density lipoprotein cholesterol; LDL-C: low-density lipoprotein cholesterol; TG: triglycerides.

*P values for paired *t*-test.

^†^ *P* values for Wilcoxon signed rank test.
